# Selecting models for the estimation of reference evapotranspiration for irrigation scheduling purposes

**DOI:** 10.1371/journal.pone.0245270

**Published:** 2021-01-11

**Authors:** Lucas Borges Ferreira, Fernando França da Cunha, Sidney Sara Zanetti

**Affiliations:** 1 Department of Agricultural Engineering, Federal University of Viçosa, Viçosa, MG, Brazil; 2 Department of Forest and Wood Sciences, Federal University of Espírito Santo, Alegre, ES, Brazil; Soil and Water Resources Institute ELGO-DIMITRA, GREECE

## Abstract

Alternative models for the estimation of reference evapotranspiration (ETo) are typically assessed using traditional error metrics, such as root mean square error (RMSE), which may not be sufficient to select the best model for irrigation scheduling purposes. Thus, this study analyzes the performance of the original and calibrated Hargreaves-Samani (HS), Romanenko (ROM) and Jensen-Haise (JH) equations, initially assessed using traditional error metrics, for use in irrigation scheduling, considering the simulation of different irrigation intervals/time scales. Irrigation scheduling was simulated using meteorological data collected in Viçosa-MG and Mocambinho-MG, Brazil. The Penman-Monteith FAO-56 equation was used as benchmark. In general, the original equations did not perform well to estimate ETo, except the ROM and HS equations used at Viçosa and Mocambinho, respectively. Calibration and the increase in the time scale provided performance gains. When applied in irrigation scheduling, the calibrated HS and JH equations showed the best performances. Even with greater errors in estimating ETo, the calibrated HS equation performed similarly or better than the calibrated JH equation, as it had errors with greater potential to be canceled during the soil water balance. Finally, in addition to using error metrics, the performance of the models throughout the year should be considered in their assessment. Furthermore, simulating the application of ETo models in irrigation scheduling can provide valuable information for choosing the most suitable model.

## Introduction

Irrigation is a very important practice to ensure good agricultural productions in arid and semiarid areas. In addition, it can contribute to reduce production risks, even in areas with reasonable rainfall levels, and can be used in greenhouse production. However, despite its benefits, irrigation should be used properly to avoid excessive or insufficient water application. In this sense, irrigation scheduling plays a key role, allowing one to provide water to different crops according to their requirements [1].

Irrigation scheduling can be performed using different approaches, but it is commonly based on reference evapotranspiration (ETo), which is typically computed using meteorological data [2–7]. ETo can be used as basis to compute the evapotranspiration of different crops. To accomplish this, a crop coefficient (Kc) and a water stress coefficient (Ks) are used to convert ETo to the evapotranspiration of a particular crop, considering its development phase and the soil water availability [6, 8].

ETo can be estimated using the Penman-Monteith FAO-56 (PM) equation, recommended by the Food and Agriculture Organization (FAO) [2, 8]. This equation performs well in different regions of the world. However, in places with low meteorological data availability, its application becomes limited, since it requires air temperature, relative humidity, solar radiation and wind speed data [9, 10].

To make it possible to estimate ETo using fewer meteorological data, several studies have evaluated the potential of empirical equations and machine learning models to estimate ETo under different meteorological data availability scenarios [10–15]. These alternative models can be important options for the estimation of ETo, however they typically have a limited performance. According to the performance of a particular model, it can be considered suitable or not for irrigation scheduling purposes.

To assess the performance of models for the estimation of ETo, traditional error metrics, such as root mean square error (RMSE), mean absolute error (MAE), mean bias error (MBE) and coefficient of determination (R^2^), are typically used [11–14, 16]. Overall, these metrics compute the dissimilarity (error) or similarity between the estimates provided by a reference model, which is commonly represented by the PM equation, and a model under evaluation. Based on a single error metric or on a set of error metrics, it is possible to define the most efficient model to estimate ETo as the one with lower errors in relation to the reference model. However, when selecting models for irrigation scheduling, the use of the strategy mentioned above do not provide a direct assessment of the performance of the models for this specific purpose.

In irrigation scheduling, irrigation frequency can have a significant influence on the performance of the models since when grouping daily ETo values in longer periods, the prediction errors may decrease. In addition, when calculating crop evapotranspiration (ETc) using a water stress coefficient (Ks), problems with ETo overestimation, which cause ETc overestimation, can be partially reduced during the soil water balance since the estimated soil water content will drop faster, promoting higher Ks reduction, which reduces the next ETc values calculated. Other important factor is the behavior of the ETo model over time. For instance, a model with random errors over time can has its errors partially canceled during the soil water balance. Finally, the rainfall distribution over the year can also impact the performance of irrigation scheduling performed with alternative ETo models. Given the dynamics of irrigation scheduling, it is highlighted that the simple use of error metrics may not be sufficient to select the best ETo model for irrigation scheduling purposes.

Despite the importance of the development of methodologies for a better assessment of models for the estimation of ETo for irrigation scheduling purposes, according to our knowledge, so far, this type of study has not been found. Thus, the objective of this study was to analyze the performance of three original and calibrated empirical equations, initially evaluated using traditional error metrics, for irrigation scheduling, considering the simulation of different irrigation intervals.

## Materials and methods

### Database

Hourly data from two automatic weather stations (2015–2017) of the Brazilian National Institute of Meteorology (INMET) located in the municipalities of Viçosa and Mocambinho, which are located in the state of Minas Gerais, Brazil, were used. Maximum and minimum air temperature, mean relative humidity, solar radiation, wind speed (10 m) and rainfall data were used. Wind speed measured at 10 m height was converted to 2 m height, as suggested by Allen et al. [8]. The hourly data were converted to a daily timescale. Days with missing data were removed. The weather stations used in this study were selected because they represent relatively different climatic conditions. The mean values of the meteorological variables used, in the periods considered to calibrate the equations (2015–2016) and to assess their performances (2017), are presented in Table 1. The database is available in Supporting information or directly from INMET (https://portal.inmet.gov.br/dadoshistoricos).

**Table 1:** Mean values of the meteorological variables used in the study.

Viçosa (Latitude: -20.77°, longitude: -42.87° and altitude: 712 m)							
Period	T_max_	T_min_	RH	Ws	Rs	P	ETo
2015–2016	28.1	16.2	78.1	0.7	16.7	1194	3.3
2017	27.4	15.4	76.7	0.6	16.5	847	3.2
Mocambinho (Latitude: -15.08°, longitude: -44.00° and altitude: 460 m)							
2015–2016	33.6	19.1	56.7	0.8	21.6	551	4.6
2017	32.9	18.3	57.1	0.9	21.9	573	4.6

T_max_—maximum air temperature (°C); T_min_—minimum air temperature (°C); RH—mean relative humidity (%); Ws—wind speed (2 m) (m s^-1^); Rs—solar radiation (MJ m^-2^ d^-1^); P—annual rainfall (mm); ETo—reference evapotranspiration (mm d^-1^).

### Irrigation scheduling—simulation configurations

To carry out irrigation scheduling, the soil water inputs (rainfall and irrigation) and output (evapotranspiration) were computed. Crop evapotranspiration (ETc) was calculated based on Eq 1, as recommended by Allen et al. [8] and Bernardo et al. [17]. Ks coefficient is used to adjust ETc for water deficit conditions. When adjusted for water deficit conditions, as considered in the present study, it is common to refer to ETc as actual evapotranspiration (ETa) or adjusted ETc. In this study, the denotation ETc was maintained.

ETc=EToKcKs (1)

where ETc—crop evapotranspiration, mm d^-1^; ETo—reference evapotranspiration, mm d^-1^; Kc—crop coefficient; Ks—water stress coefficient.

ETo was obtained using different equations, which are presented later. Ks was calculated based on Eq 2 [17].

$$Ks=\frac{ln\;(SWC+1)}{ln\;(TAW+1)}$$ (2)

Where SWC—soil water content, mm; TAW—total available water, mm.

$$TAW=\frac{\left(FC-PWP\right)}{10}BD\;z$$ (3)

Where TAW—total available water, mm; FC—field capacity, % (water mass over dry soil mass); PWP—permanent wilting point, % (water mass over dry soil mass); BD—soil bulk density, g cm^-3^; z—effective rooting depth, cm.

Once ETc has been obtained, the soil water balance was computed based on Eq 4. The initial value of the soil water content (SWC) was equal to TAW. Effective rainfall (rainfall stored in the root zone) was considered equal to total rainfall, if total rainfall does not exceed the current soil water deficit (TAW–SWC), or equal to the current soil water deficit, otherwise.

$${SWC}_{i}={SWC}_{i-1}-ETc+Pe+I$$ (4)

Where SWC_i_—soil water content on the current day, mm; SWC_i-1_—soil water content on the previous day, mm; ETc—crop evapotranspiration, mm; Pe—effective rainfall, mm; I—net irrigation depth, mm.

Knowing the current SWC, irrigation was computed in order to return SWC to field capacity. Thus, net irrigation depth was obtained by subtracting SWC from TAW (TAW—SWC). The parameters used for the simulations were as follows: field capacity (FC) = 30%, permanent wilting point (PWP) = 15%, soil bulk density (BD) = 1.1 g cm^-3^, effective rooting depth (z) = 20 cm, and crop coefficient (Kc) = 1.1. Fixed irrigation intervals (1, 2, 4, 6 and 8 days) and variable irrigation intervals were considered. For variable irrigation intervals, the critical minimum soil water content was defined as 50% of TAW, which is considered by using a soil water depletion fraction for no stress (p), also called soil water availability factor (f), equal to 0.5. It is assumed that below this water content the crop begins to be affected by water deficit. To prevent the soil water content from exceeding the aforementioned critical minimum limit, irrigation was carried out when the soil water content was 40% below TAW. The simulations were performed using data from the year 2017, with data from 2015–2016 reserved to calibrate the empirical equations.

### Estimation of reference evapotranspiration

Daily ETo estimated using the PM equation (Eq 5) was employed as the standard method for calibration and evaluation of the empirical equations. All procedures necessary to calculate ETo were performed according to the recommendations of Allen et al. [8]. Although the PM equation is also subject to errors, it has good reliability and can be used as a standard for the development and calibration of other models [8, 9].

$$ETo=\frac{0.408\;\Delta (R_{n}‐G)+\gamma \frac{900}{T_{\mathrm{mean}}+273}u_{2}(e_{s}‐e_{a})}{\Delta +\gamma (1+0.34\;u_{2})}$$ (5)

where ETo—reference evapotranspiration, mm d^-1^; R_n_—net solar radiation, MJ m^-2^ day^-1^; G—soil heat flux, MJ m^-2^ day^-1^ (considered to be null for daily estimates); T_mean_—daily mean air temperature, °C, u_2_—wind speed at a 2 m height, m s^-1^; e_s_—saturation vapor pressure, kPa; e_a_—actual vapor pressure, kPa; Δ—slope of the saturation vapor pressure function, kPa °C^-1^; and γ—psychrometric constant, kPa °C^-1^.

ETo was also estimated using the empirical equations shown in Table 2.

**Table 2:** Empirical equations used in the study.

Name / Inputs	Equation	Reference
Hargreaves-Samani (T)	$\mathrm{ET}o=0.0023R_{a}(T_{\mathrm{mean}}+17.8){\left(T_{\max}‐T_{\min}\right)}^{0.5}$	[18]
Romanenko (T, RH)	$\mathrm{ET}o=0.00006{\left(25+T_{\mathrm{me}\mathrm{an}}\right)}^{2}(100-RH)$	[19]
Jensen-Haise (T, R_s_)	$\mathrm{ET}o=0.408R_{s}(0.0252T_{\mathrm{me}\mathrm{an}}+0.078)$	[20]

T—air temperature, °C; RH–mean relative humidity, %; R_a_—extraterrestrial radiation, mm d^-1^; T_max_—maximum air temperature, °C; Tmin—minimum air temperature, °C; T_mean_—mean air temperature ([T_max_+ T_min_]/2), °C; R_s_—solar radiation, MJ m^-2^ d^-1^.

To adjust the empirical equations to the local climate conditions, they were calibrated based on simple linear regression, as recommended by Allen et al. [8], using data from 2015 to 2016. For this, daily ETo values estimated by the equation to be calibrated were used as the independent variable and ETo values estimated by the PM equation were used as the dependent variable. The intercept (a) and slope (b) values were used as calibration parameters, according to the following equation. The values obtained for the calibration parameters “a” and “b” are presented in Table 3.

**Table 3:** Calibration parameters obtained for the different empirical equations evaluated at Viçosa and Mocambinho stations.

	Viçosa		Mocambinho	
Equation	a	b	a	b
Hargreaves-Samani	-0.76	0.90	-0.46	0.92
Romanenko	0.79	0.83	2.26	0.33
Jensen-Haise	0.35	0.66	0.51	0.61

$${ETo}_{cal}=a+b(ETo)$$ (6)

where ETo_cal_—calibrated reference evapotranspiration, mm d^-1^; “a” and “b”—calibration parameters; ETo—reference evapotranspiration estimated by the original (non-calibrated) empirical equation, mm d^-1^.

### Performance comparison criteria

The performance of the empirical equations for the estimation of ETo was evaluated using data from the year 2017, the same period considered for irrigation scheduling. For that, ETo obtained with the PM equation was used as standard. The empirical equations were evaluated in different time scales (1, 2, 4, 6 and 8 days) by summing daily estimates. The error metrics listed below were used. Except for coefficient of determination (R^2^), normalized values of each error metric were calculated. For that, the error metrics values were divided by the mean of the analyzed variable (mean of the observed values). For time scales equal or greater than 2 days, the error metrics, except for R^2^, were divided by the time scale in order to keep the unit mm d^-1^.

$$RMSE=\sqrt{\frac{1}{n}\sum {\left(P_{i}-O_{i}\right)}^{2}}$$ (7)

$$MAE=\frac{1}{n}\sum \left|P_{i}-O_{i}\right|$$ (8)

$$MBE=\frac{1}{n}\sum \left(P_{i}-O_{i}\right)$$ (9)

$$R^{2}={\left[\frac{\sum {(P}_{i}-\bar{P}){(O}_{i}-\bar{O})}{\sqrt{{(\sum {(P}_{i}-\bar{P})}^{2}){(\sum {(O}_{i}-\bar{O})}^{2})}}\right]}^{2}$$ (10)

where RMSE—root mean square error, mm d^-1^; MAE—mean absolute error, mm d^-1^; MBE—mean bias error, mm d^-1^; R^2^—coefficient of determination; P_i_—predicted value, mm d^-1^; O_i_—observed value, mm d^-1^; $\bar{P}$—mean of the predicted values, mm d^-1^; $\bar{O}$—mean of the observed values, mm d^-1^; n—number of data pairs.

**Fig 1:**
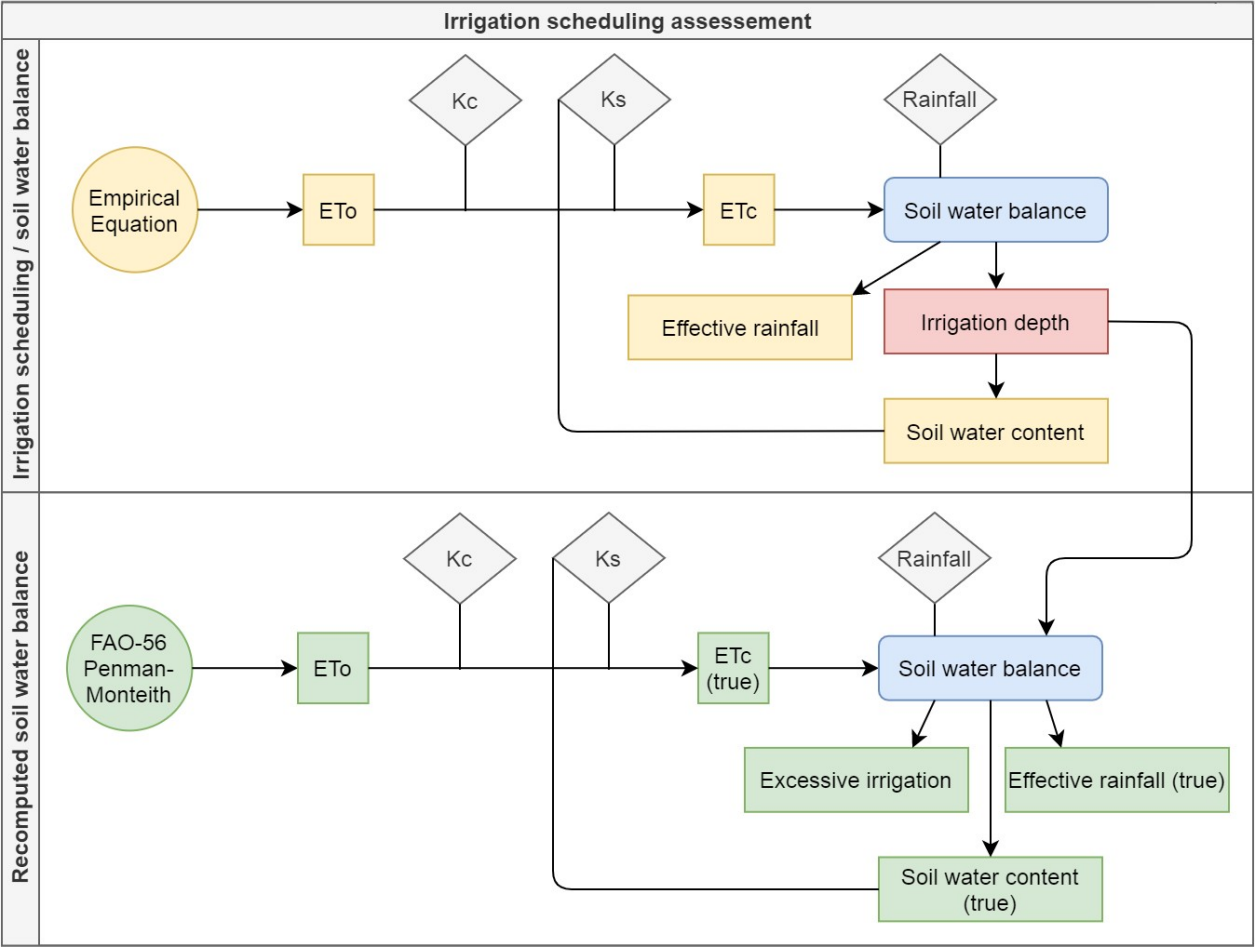
Proposed methodology to assess the performance of different empirical equations for irrigation scheduling.

To assess the performance of the equations in the simulated irrigation scheduling, total ETc, total net irrigation depth and total effective rainfall estimated when using each equation were compared. In addition, after the end of the irrigation scheduling carried out with each empirical equation, the soil water balance was recomputed considering the irrigations recommended over the management period and ETc recalculated using ETo obtained with the PM equation (Fig 1). All the computations were performed in a daily basis. ETc and effective rainfall obtained in the recomputed soil water balance were denoted as ETc (true) and Pe (true), respectively. This procedure was performed to assess the real performance of the irrigation scheduling carried out with the different empirical equations. In this way, it is possible to analyze ETc that actually occurred during the management period and check the occurrence of irrigation excesses or deficits when using the different empirical equations to schedule irrigation.

Based on the recomputed soil water balance (Fig 1), the irrigation excesses and deficits occurred during the management period were calculated. Irrigation excesses were computed as the sum of the net irrigation depths that resulted in soil water contents that exceeded field capacity. To compute irrigation deficits for the simulations with fixed irrigation intervals, deficit was considered as the reduction of ETc (true) observed for each empirical equation in relation to ETc observed when using the PM equation to schedule irrigation. It was done because water deficit promotes reductions in ETc, which is related to a worse crop development. For irrigation scheduling with variable irrigation intervals, the occurrence of deficits was computed as the sum of the soil water content deficits in relation to the critical minimum water content considered (50% of TAW, f = 0.5). Two classes of deficit were defined: (i) cases in which deficits were equivalent to 0.5<f≤0.6 (weak deficit), and (ii) f>0.6 (moderate to strong deficit). These deficits were calculated according to Eqs 11 and 12.

$$Deficit(0.5<f\leq 0.6)=\sum \left({CL}_{1}-{SWC}_{i}\right),\;for\;{CL}_{2}\leq {SWC}_{i}<{CL}_{1}$$ (11)

$$Deficit(f>0.6)=\sum \left({CL}_{1}-{SWC}_{i}\right),\;for\;{SWC}_{i}<{CL}_{2}$$ (12)

$${CL}_{1}=0.5TAW$$ (13)

$${CL}_{2}=0.4TAW$$ (14)

where CL_1_—critical limit soil water content referring to f = 0.5, mm; CL_2_—critical limit soil water content referring to f = 0.6, mm; SWC_i_—soil water content value, mm; TAW—total available water, mm.

## Results and discussion

### Estimation of ETo

Among the non-calibrated equations, the Romanenko equation (ROM) had the best performance for the estimation of ETo at Viçosa, with lower RMSE and MAE values in the various time scales considered (Table 4). This equation was followed by the Jensen-Haise (JH) equation and the Hargreaves-Samani (HS) equation, in that order. However, after calibration, the ROM equation exhibited the worst performance. The best performance was obtained by the JH equation, followed by the HS equation.

**Table 4:** Performance of the original and calibrated HS, ROM and JH equations for different time scales at Viçosa. Values in parentheses indicate the normalized error metrics.

Equation	Scale (d)	RMSE (mm d^-1^)	MAE (mm d^-1^)	MBE (mm d^-1^)	R^2^
HS	1	1.30 (41%)	1.21 (38%)	1.20 (38%)	0.84
	2	1.26 (40%)	1.20 (38%)	1.20 (38%)	0.89
	4	1.24 (39%)	1.20 (38%)	1.20 (38%)	0.92
	6	1.22 (39%)	1.19 (38%)	1.19 (38%)	0.94
	8	1.22 (39%)	1.20 (38%)	1.20 (38%)	0.96
HS_cal	1	0.47 (15%)	0.36 (11%)	0.01 (0%)	0.84
	2	0.37 (12%)	0.28 (9%)	0.01 (0%)	0.89
	4	0.29 (9%)	0.21 (7%)	0.00 (0%)	0.92
	6	0.25 (8%)	0.19 (6%)	0.00 (0%)	0.94
	8	0.18 (6%)	0.13 (4%)	0.00 (0%)	0.96
ROM	1	0.72 (23%)	0.56 (18%)	-0.10 (-3%)	0.68
	2	0.65 (20%)	0.52 (16%)	-0.09 (-3%)	0.70
	4	0.60 (19%)	0.50 (16%)	-0.09 (-3%)	0.69
	6	0.59 (19%)	0.48 (15%)	-0.09 (-3%)	0.67
	8	0.58 (18%)	0.49 (15%)	-0.09 (-3%)	0.66
ROM_cal	1	0.70 (22%)	0.59 (19%)	0.17 (6%)	0.68
	2	0.64 (20%)	0.55 (17%)	0.18 (6%)	0.70
	4	0.60 (19%)	0.53 (17%)	0.18 (6%)	0.69
	6	0.59 (19%)	0.53 (17%)	0.18 (6%)	0.67
	8	0.58 (18%)	0.52 (17%)	0.18 (6%)	0.66
JH	1	1.23 (39%)	1.09 (34%)	1.08 (34%)	0.97
	2	1.20 (38%)	1.08 (34%)	1.08 (34%)	0.98
	4	1.17 (37%)	1.08 (34%)	1.08 (34%)	0.98
	6	1.16 (37%)	1.08 (34%)	1.08 (34%)	0.98
	8	1.15 (36%)	1.08 (34%)	1.08 (34%)	0.98
JH_cal	1	0.20 (6%)	0.15 (5%)	-0.02 (-1%)	0.97
	2	0.18 (6%)	0.14 (5%)	-0.02 (-1%)	0.98
	4	0.17 (5%)	0.14 (4%)	-0.02 (-1%)	0.98
	6	0.17 (5%)	0.14 (4%)	-0.02 (-1%)	0.98
	8	0.16 (5%)	0.14 (4%)	-0.02 (-1%)	0.98

HS—Hargreaves-Samani; ROM—Romanenko; JH—Jensen-Haise. “_cal” indicates the calibrated version of an equation.

At Mocambinho, the HS equation showed the best performance among the non-calibrated equations, followed by the JH and ROM equations, in that order (Table 5). After calibration, as for Viçosa, the JH equation showed the best performance, followed by the HS and ROM equations. Possibly the HS equation obtained the best performance among the non-calibrated equations because it was developed for a dry climate region (semiarid) [21], such as Mocambinho.

**Table 5:** Performance of the original and calibrated HS, ROM and JH equations for different time scales at Mocambinho. Values in parentheses indicate the normalized error metrics.

Equation	Scale (d)	RMSE (mm d^-1^)	MAE (mm d^-1^)	MBE (mm d^-1^)	R^2^
HS	1	1.00 (22%)	0.87 (19%)	0.82 (18%)	0.76
	2	0.95 (21%)	0.83 (18%)	0.82 (18%)	0.81
	4	0.90 (20%)	0.82 (18%)	0.82 (18%)	0.86
	6	0.89 (19%)	0.82 (18%)	0.82 (18%)	0.88
	8	0.88 (19%)	0.82 (18%)	0.82 (18%)	0.88
HS_cal	1	0.57 (13%)	0.43 (9%)	-0.07 (-2%)	0.76
	2	0.48 (11%)	0.37 (8%)	-0.07 (-2%)	0.81
	4	0.39 (9%)	0.32 (7%)	-0.07 (-2%)	0.86
	6	0.35 (8%)	0.28 (6%)	-0.07 (-2%)	0.88
	8	0.33 (7%)	0.27 (6%)	-0.07 (-2%)	0.88
ROM	1	2.55 (56%)	2.19 (48%)	2.04 (45%)	0.39
	2	2.52 (55%)	2.19 (48%)	2.05 (45%)	0.39
	4	2.49 (55%)	2.15 (47%)	2.05 (45%)	0.38
	6	2.45 (54%)	2.13 (47%)	2.05 (45%)	0.36
	8	2.43 (53%)	2.12 (46%)	2.05 (45%)	0.34
ROM_cal	1	0.90 (20%)	0.77 (17%)	-0.11 (-2%)	0.39
	2	0.85 (19%)	0.73 (16%)	-0.11 (-2%)	0.39
	4	0.80 (18%)	0.69 (15%)	-0.11 (-2%)	0.38
	6	0.79 (17%)	0.67 (15%)	-0.11 (-2%)	0.36
	8	0.78 (17%)	0.67 (15%)	-0.11 (-2%)	0.34
JH	1	2.10 (46%)	1.94 (43%)	1.94 (43%)	0.91
	2	2.07 (46%)	1.94 (43%)	1.94 (43%)	0.91
	4	2.05 (45%)	1.94 (43%)	1.94 (43%)	0.92
	6	2.04 (45%)	1.94 (43%)	1.94 (43%)	0.92
	8	2.03 (45%)	1.94 (43%)	1.94 (43%)	0.92
JH_cal	1	0.36 (8%)	0.26 (6%)	-0.07 (-2%)	0.91
	2	0.33 (7%)	0.25 (5%)	-0.07 (-2%)	0.91
	4	0.30 (7%)	0.23 (5%)	-0.07 (-2%)	0.92
	6	0.28 (6%)	0.21 (5%)	-0.07 (-2%)	0.92
	8	0.28 (6%)	0.20 (4%)	-0.07 (-2%)	0.92

HS—Hargreaves-Samani; ROM—Romanenko; JH—Jensen-Haise. “_cal” indicates the calibrated version of an equation.

By increasing the time scale, there were performance gains for all the equations at both municipalities considered, with reductions in the error metrics (RMSE, MAE and MBE) and increase in R^2^. This is because part of the errors in daily estimates can be canceled when considering longer time periods.

All the non-calibrated equations evaluated, with exception for the ROM equation used at Viçosa, showed relatively high MBE values at both studied locations, which indicates that there was a systemic overestimation of ETo. These equations obtained only small performance gains with the increase of the time scale. Furthermore, they did not reach RMSE and MAE values as low as those obtained by the calibrated equations, which showed a low general tendency to overestimate or underestimate ETo (low MBE absolute values).

Calibration promoted large reductions in RMSE and MAE values. After calibration, the equations with higher R^2^ values, with emphasis on the JH equation, even with high RMSE and MAE values before calibration, exhibited low errors. It should be noted that equations with good structure, which can adequately map the relationship between the input and output variables, reaching high R^2^ values, can be benefited by calibration [16].

Based on the metrics presented in Tables 4 and 5, one can easily rank the performance of the models, identifying those with the highest performances. However, it can still be difficult to infer whether a particular model is suitable or not for irrigation scheduling purposes.

### Irrigation scheduling

The results of the irrigation scheduling simulations with fixed irrigation intervals for Viçosa and Mocambinho are shown in Tables 6 and 7, respectively. The increase in irrigation intervals promoted, in all cases, reductions in ETc values and in the total net irrigation depths applied. The decrease in ETc occurs due to the larger reductions in the soil moisture promoted by larger irrigation intervals, which reduces Ks values and, consequently, ETc. The reduction in the net irrigation depths occurs due to the reduction in ETc and due to the increase in effective rainfall, as seen in Tables 6 and 7. Longer irrigation intervals promote greater use of rainfall (i.e., more rainwater is stored in the root zone) because they increase the chance of soil having less moisture, in relation to shorter irrigation intervals, when rainfall reaches the soil.

**Table 6:** Information on the irrigation scheduling carried out at Viçosa with the PM equation and original and calibrated empirical equations considering different irrigation intervals (II). All the variables, except for II, are expressed in mm.

Equation	II (d)	ETc	ETc (true)	NID	Pe	Pe (true)	Deficit	Excess
PM	1	1204	1204	1014	189	189	-	-
	2	1186	1186	958	229	229	-	-
	4	1150	1150	863	286	286	-	-
	6	1114	1114	767	348	348	-	-
	8	1074	1074	697	377	377	-	-
HS	1	1663	1204	1402	262	189	0	387
	2	1631	1186	1318	314	229	0	360
	4	1561	1150	1187	374	286	0	324
	6	1485	1114	1059	426	348	0	292
	8	1393	1074	930	463	377	0	233
HS_cal	1	1206	1192	999	207	214	12	22
	2	1189	1175	941	248	253	11	19
	4	1154	1138	849	305	304	11	14
	6	1120	1105	759	361	358	9	12
	8	1082	1063	686	396	385	11	8
ROM	1	1166	1184	1005	160	256	20	77
	2	1149	1168	947	201	289	18	69
	4	1113	1132	853	260	340	17	60
	6	1077	1094	761	316	387	20	53
	8	1035	1054	692	343	407	20	45
ROM_cal	1	1270	1191	1088	182	232	13	129
	2	1251	1174	1027	224	270	12	122
	4	1211	1139	928	282	321	11	111
	6	1170	1101	828	342	373	13	100
	8	1122	1061	757	365	398	13	93
JH	1	1614	1204	1391	223	189	0	377
	2	1581	1186	1309	271	229	0	352
	4	1508	1150	1166	342	286	0	303
	6	1425	1114	1029	395	348	0	263
	8	1331	1074	899	431	377	0	202
JH_cal	1	1196	1197	1011	185	209	6	23
	2	1179	1180	955	224	247	6	22
	4	1143	1144	864	280	300	6	20
	6	1109	1109	769	340	359	5	19
	8	1070	1068	700	370	385	6	17

NID—total net irrigation depth; Pe—effective rainfall; Deficit—ETc deficit in relation to ETc obtained in the irrigation scheduling performed with the PM equation; Excess—excessive irrigation; ETc (true) and Pe (true)—ETc and Pe recalculated using ETo obtained with the PM equation. All the variables are expressed in mm. HS—Hargreaves-Samani; ROM—Romanenko; JH—Jensen-Haise. “_cal” indicates the calibrated version of an equation.

**Table 7:** Information on the irrigation scheduling carried out at Mocambinho with the PM equation and original and calibrated empirical equations considering different irrigation intervals (II). All the variables, except for II, are expressed in mm.

Equation	II (d)	ETc	ETc (true)	NID	Pe	Pe (true)	Deficit	Excess
PM	1	1809	1809	1651	158	158	-	-
	2	1768	1768	1550	213	213	-	-
	4	1680	1680	1456	219	219	-	-
	6	1569	1569	1292	271	271	-	-
	8	1423	1423	1157	259	259	-	-
HS	1	2133	1808	1935	199	158	1	285
	2	2078	1768	1815	256	213	0	265
	4	1954	1680	1683	264	219	0	227
	6	1788	1569	1459	320	271	0	166
	8	1571	1423	1259	301	259	0	102
HS_cal	1	1781	1764	1606	175	179	44	21
	2	1742	1726	1508	228	228	43	16
	4	1661	1641	1419	236	230	39	15
	6	1559	1538	1266	287	277	31	12
	8	1428	1405	1147	274	261	18	11
ROM	1	2619	1807	2465	154	163	2	823
	2	2524	1766	2310	209	214	2	764
	4	2277	1678	2050	214	219	2	597
	6	1917	1567	1621	279	271	3	332
	8	1572	1419	1295	257	259	4	142
ROM_cal	1	1765	1744	1609	156	235	65	106
	2	1728	1704	1521	203	270	65	101
	4	1647	1622	1434	209	263	58	91
	6	1548	1519	1288	255	297	50	80
	8	1420	1392	1170	245	273	31	60
JH	1	2580	1809	2388	193	158	0	737
	2	2493	1768	2230	254	213	0	680
	4	2279	1680	2006	264	219	0	550
	6	1953	1569	1605	328	271	0	312
	8	1649	1423	1321	308	259	0	164
JH_cal	1	1780	1766	1625	155	171	43	29
	2	1741	1727	1525	211	223	41	27
	4	1657	1644	1436	216	226	37	24
	6	1553	1542	1279	269	276	27	19
	8	1418	1411	1155	255	260	13	13

NID—total net irrigation depth; Pe—effective rainfall; Deficit—ETc deficit in relation to ETc obtained in the irrigation scheduling performed with the PM equation; Excess—excessive irrigation; ETc (true) and Pe (true)—ETc and Pe recalculated using ETo obtained with the PM equation. All the variables are expressed in mm. HS—Hargreaves-Samani; ROM—Romanenko; JH—Jensen-Haise. “_cal” indicates the calibrated version of an equation.

Among the non-calibrated equations, only the ROM equation used at Viçosa obtained total net irrigation depth close to that obtained with the PM equation. In all other cases, irrigation was overestimated. Thus, such equations promoted excessive water application, increasing the soil moisture above field capacity, as seen in the “Excess” column of Tables 6 and 7. However, after calibration, all the equations obtained total net irrigation depths close to those obtained when using the PM equation. Such behaviors corroborate the reductions in MBE absolute values observed for the estimation of ETo (Tables 4 and 5).

Although the calibrated equations obtained total net irrigation depths close to those obtained using the PM equation, it does not mean that they had the same performance of the PM equation. It may happen that, over the year, the overestimated irrigations have been compensated for the underestimated irrigations, canceling the errors. Thus, irrigation scheduling must be evaluated considering its dynamics over time.

To analyze the performance of the equations considering their time dynamics, it is possible to evaluate the occurrence of excessive water applications, as well as reductions of ETc under adequate irrigation conditions (i.e., irrigation scheduling using the PM equation) in relation to ETc observed under lower water application (i.e., irrigation scheduling using alternative equations). In this sense, although most of the calibrated equations resulted in total net irrigation depths close to those calculated with the PM equation, there were both irrigation underestimation and overestimation during the period evaluated, as shown in columns “Deficit” and “Excess” in Tables 6 and 7. However, after calibrating the equations, there were, in general, large reductions in the excessive water applications. On the other hand, the calibrated equations promoted certain irrigation deficits, slightly reducing total ETc (true) in relation to that observed when scheduling irrigation with the PM equation. For both study sites, the calibrated HS and JH equations were the best options, promoting low excessive water applications and only small reductions in ETc (true).

When scheduling irrigation using variable irrigation intervals, a critical soil water content is adopted to prevent the crop from suffering water deficit. Thus, it is necessary that the current soil water content is always above or, at most, slightly below the critical minimum limit considered. Thus, alternative models for the estimation of ETo must be able to provide sufficiently reliable ETo estimates to meet the condition described above. The results of the irrigation scheduling simulations with variable irrigation intervals are shown in Table 8.

**Table 8:** Information on the irrigation scheduling carried out at Viçosa and Mocambinho with the PM equation and original and calibrated empirical equations using variable irrigation intervals. All the variables are expressed in mm.

Station	Equation	ETc	ETc (true)	NID	Pe	Pe (true)	Deficit (0.5<f≤0.6)	Deficit (f>0.6)	Excess
Viçosa	PM	1142	1142	866	276	276	5 (7 days)	0	-
	HS	1580	1162	1252	327	249	0	0	340 (81 days)
	HS_cal	1144	1127	816	327	320	29 (24 days)	0	10 (14 days)
	ROM	1106	1122	835	271	347	36 (23 days)	52 (11 days)	61 (33 days)
	ROM_cal	1204	1133	916	288	324	33 (21 days)	18 (4 days)	108 (41 days)
	JH	1535	1162	1224	311	262	0	0	324 (78 days)
	JH_cal	1134	1134	854	280	299	22 (16 days)	5 (1 day)	19 (26 days)
Moc.	PM	1713	1713	1448	256	256	37 (31 days)	0	-
	HS	2029	1736	1739	284	233	5 (12 days)	0	242 (107 days)
	HS_cal	1687	1670	1411	267	261	80 (47 days)	107 (20 days)	12 (14 days)
	ROM	2498	1751	2255	238	233	11 (9 days)	0	742 (127 days)
	ROM_cal	1677	1653	1433	240	298	66 (42 days)	247 (38 days)	91 (42 days)
	JH	2460	1754	2198	254	213	0	0	663 (132 days)
	JH_cal	1686	1672	1431	249	259	64 (46 days)	153 (25 days)	24 (38 days)

NID—total net irrigation depth; Pe—effective rainfall; Deficit (0.5<f≤0.6) and Deficit (f>0.6)—sum of soil water content deficits in relation to the critical level (50% of TAW, f = 0.5) in the cases of deficits equivalent to 0.5<f≤0.6 and f>0.6, respectively (values in parentheses indicate the number of days that the deficits occurred); Excess—excessive irrigation (values in parentheses indicate the number of days that the irrigation excesses occurred); ETc (true) and Pe (true)—ETc and Pe recalculated using ETo obtained with the PM equation. All the variables are expressed in mm. HS—Hargreaves-Samani; ROM—Romanenko; JH—Jensen-Haise. “_cal” indicates the calibrated version of an equation.

As previously observed, among the non-calibrated equations, only the ROM equation used at Viçosa obtained total net irrigation depth close to that obtained with the PM equation. In the other cases, the total net irrigation depths were much higher than those calculated with the PM equation. After calibrations, there were, in general, reductions in the irrigation excesses.

In relation to the irrigation deficits over the period evaluated, accumulated deficits in relation to the critical soil water content (f = 0.5) were computed in two classes: (i) cases in which deficits were equivalent to 0.5<f≤0.6 (weak deficit), and (ii) f>0.6 (moderate to strong deficit). Even using the PM equation, there were some weak deficit events (0.5<f≤0.6). This behavior is expected because even though the soil has not reached the limit water content for irrigation (in this study, irrigation was carried out when the soil water content was 40% below TAW) on a particular day, it is possible that, on the next day, the soil water content is already below the critical limit adopted (50% of TAW). However, it is expected that this level of stress, which remains for a short period and is of low intensity, does not cause significant damage to the crops.

**Fig 2:**
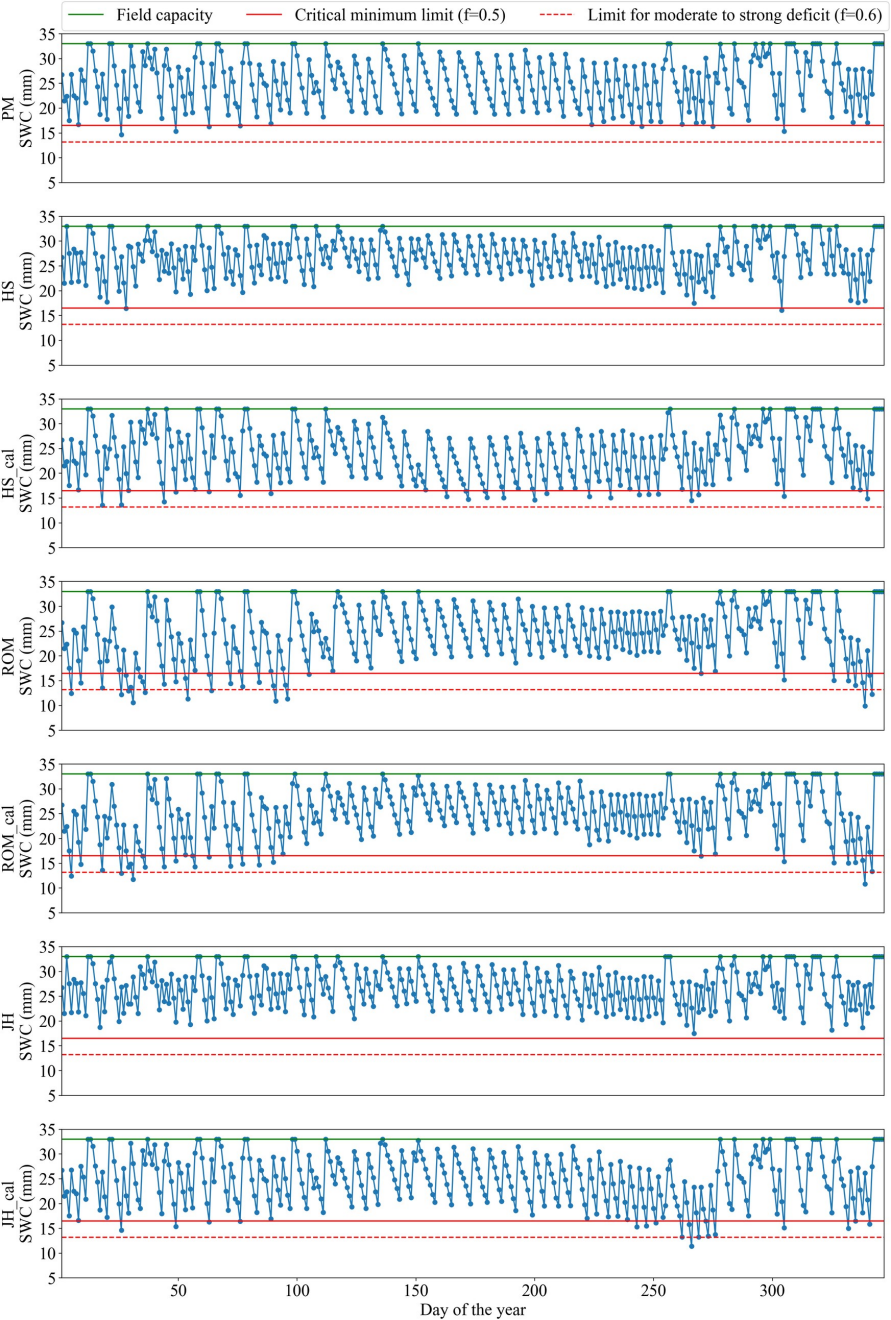
Soil water content (SWC) for irrigation scheduling with variable irrigation intervals at Viçosa using the PM equation and the original and calibrated HS, ROM and JH equations. SWC values presented were recalculated at the end of the irrigation scheduling (performed with the empirical equations) using ETo obtained by the PM equation. Days are numbered according to their order throughout the test year.

**Fig 3:**
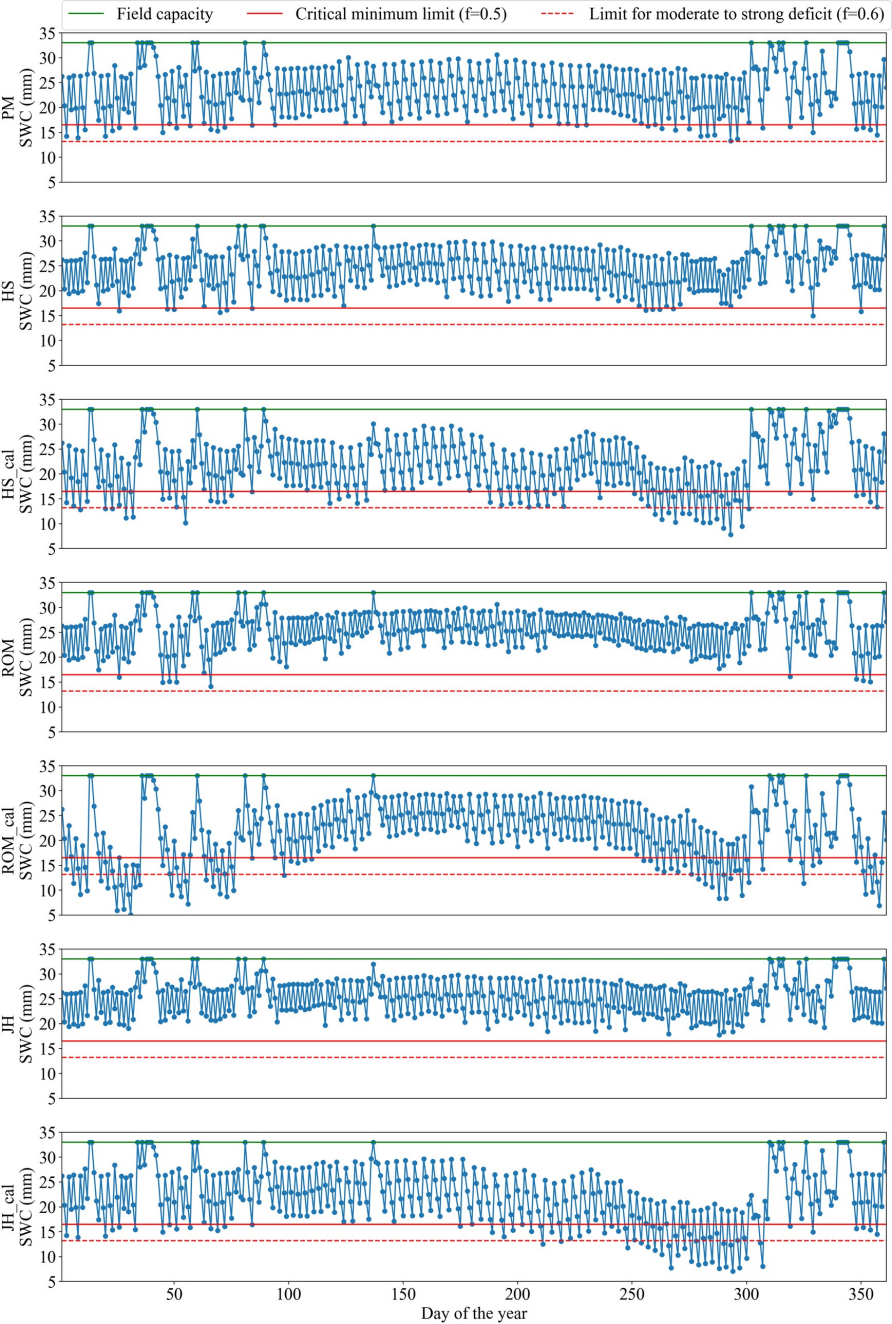
Soil water content (SWC) for irrigation scheduling with variable irrigation intervals at Mocambinho using the PM equation and the original and calibrated HS, ROM and JH equations. SWC values presented were recalculated at the end of the irrigation scheduling (performed with the empirical equations) using ETo obtained by the PM equation. Days are numbered according to their order throughout the test year.

At Viçosa, the calibrated HS and JH equations performed the best, with similar performance to each other. For Mocambinho, these equations also obtained the best performances; however, the calibrated HS equation was slightly better than the calibrated JH equation since it had lower moderate to strong deficits (f>0.6) and lower irrigation excesses. These behaviors partially contradict the results obtained when directly evaluating the equations for the estimation of ETo (Tables 4 and 5), since the calibrated JH equation was considered better than the calibrated HS equation in all the studied scenarios. To better assess the irrigation scheduling carried out with the different equations for the estimation of ETo, the soil water content behaviors during the evaluation period at Viçosa and Mocambinho are shown in Figs 2 and 3, respectively. After the end of the irrigation scheduling simulations with each empirical equation, the soil water contents were recalculated based on ETo obtained with the PM equation, as shown in Fig 1. The information presented in Figs 2 and 3 is referring to these recalculated water contents. On the days when there was irrigation, the water contents presented refer to the moment before irrigation.

**Fig 4:**
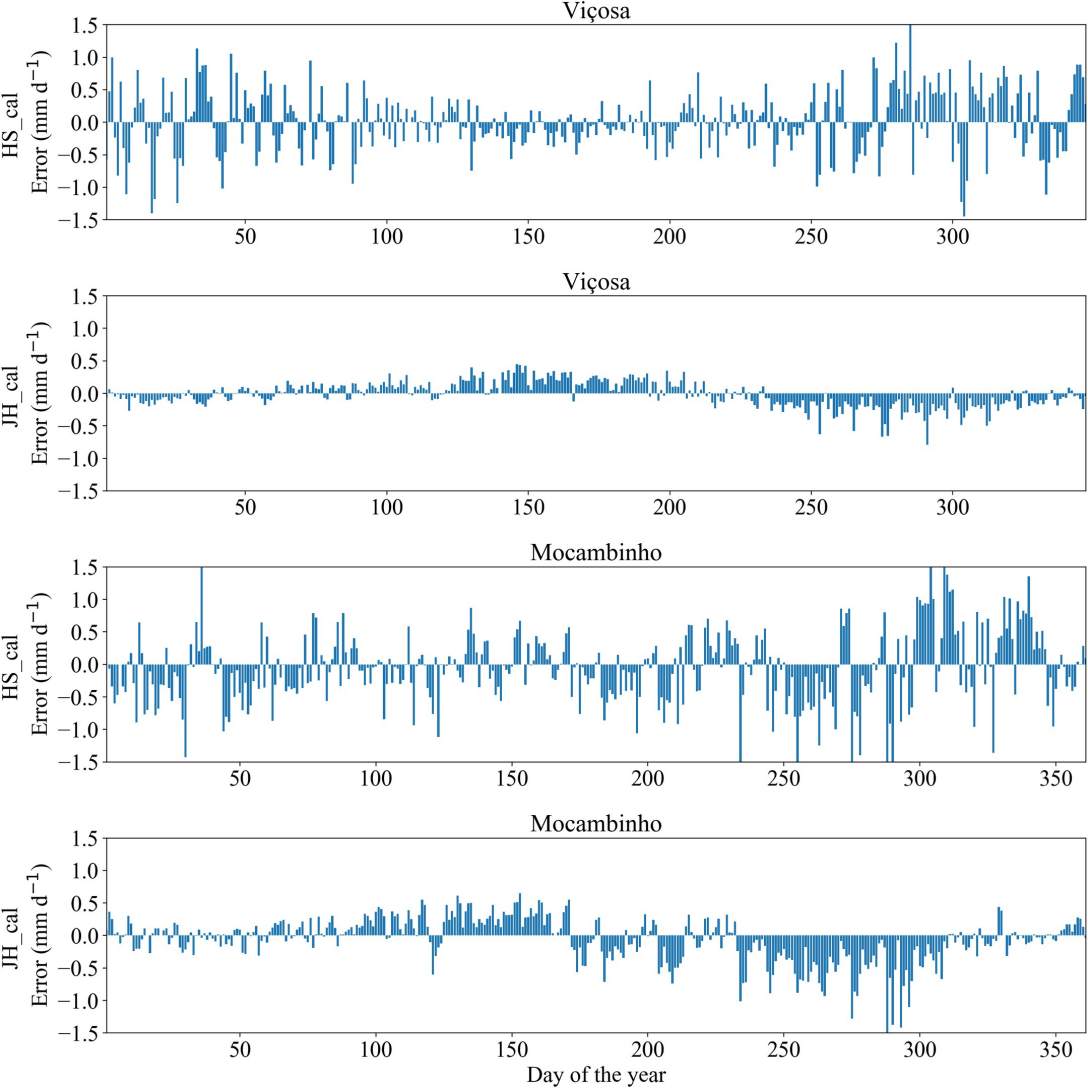
Deviation of ETo estimated by the calibrated Hargreaves-Samani (HS) and Jensen-Haise (JH) equations in relation to ETo obtained with the PM equation at Viçosa and Mocambinho. Days are numbered according to their order throughout the test year.

At Viçosa, both the calibrated HS and JH equations promoted only small water deficits below the critical limit (Fig 2). At Mocambinho, when using the calibrated HS and JH equations, the soil water content falls considerably in the period of 250 to 300 days, especially for the calibrated JH equation (Fig 3). Even though the calibrated JH equation presented better metrics than the calibrated HS equation for the estimation of ETo at Mocambinho (Table 5), this equation had continuous ETo underestimations in the period around 250–300 days (Fig 4). On the other hand, the calibrated HS equation, despite showing, in general, greater deviations in relation to ETo obtained with the PM equation, had more alternate ETo underestimates and overestimates, which contributes to partially cancel the errors occurred during the irrigation scheduling period. Similar behavior was observed for Viçosa (Fig 4). It is also worth mentioning that in places with high rainfall levels, problems with ETo underestimation tend to be reduced.

Finally, in addition to evaluating alternative models for the estimation of ETo using error metrics such as RMSE, MAE, MBE and R^2^, it is also important to analyze their behavior throughout the year. Furthermore, the simulation of the use of these models for irrigation scheduling can help in choosing the best model. Future studies could address the development of software, including integration with crop models, for simulating the use of models for the estimation of ETo for irrigation scheduling. Thus, it would be possible to evaluate the performance of the models considering more specific scenarios of interest. It could be considered, for example, the application of an empirical equation only at a certain period of the year, irrigation of crops with different cycles, and different types of soil, among other factors. Another important issue to be considered in future studies and a limitation of the present study is the use of real field data as benchmark, such as eddy covariance and/or soil water content measurements.

## Conclusions

Alternative models for the estimation of ETo are typically assessed using error metrics. However, the model with the best metrics for the estimation of ETo may not be the best option to be used for irrigation scheduling. Despite the importance of the development of methodologies for a better assessment of the performance of models for the estimation of ETo for irrigation scheduling purposes, according to our knowledge, so far, this type of study has not been found. Thus, this study analyzes the performance of three original and calibrated empirical equations, initially assessed using traditional error metrics, for irrigation scheduling, considering the simulation of different irrigation intervals. Two study sites, Viçosa-MG and Mocambinho-MG, Brazil, were used.

In general, the original empirical equations did not perform well for the estimation of ETo, with the exception of the Romanenko and Hargreaves-Samani equations used at Viçosa and Mocambinho, respectively. Calibration promoted performance gains, reducing the tendency of the equations to overestimate ETo. The increase in the time scale also led to reductions in estimation errors.

When used for irrigation scheduling, the calibrated Hargreaves-Samani and Jensen-Haise equations showed the best performances in both Viçosa and Mocambinho stations. Even with greater errors when estimating ETo, the calibrated Hargreaves-Samani equation performed similarly or better than the calibrated Jensen-Haise equation, as it had errors with greater potential to be canceled during the soil water balance. The results obtained are dependent of the climate conditions of the study site, thus, the performance of the equations can be very different in areas with different climatic conditions.

Finally, it is suggested that the assessment of models for the estimation of ETo for use in irrigation scheduling, in addition to using traditional error metrics, consider the performance of the models throughout the year. Furthermore, simulating the application of the models in irrigation scheduling can provide valuable information for choosing the most suitable option.

## Supporting information

S1 File Meteorological data from Viçosa weather station. (XLSX)

S2 File Meteorological data from Mocambinho weather station. (XLSX)

S1 Text (TXT)
